# Supplementary material for: A Multi-Gene Signature of Non-Muscle-Invasive Bladder Cancer Identifies Patients Who Respond to Immunotherapies Including Bacillus Calmette–Guérin and Immune Checkpoint Inhibitors
Source: Int J Mol Sci. 2024 Mar 28;25(7):3800. doi: 10.3390/ijms25073800 (PMC11012101; doi:10.3390/ijms25073800)
Supplement: Supplementary file 1 [file ijms-25-03800-s001.zip › ijms-2925111-supplementary.pdf]

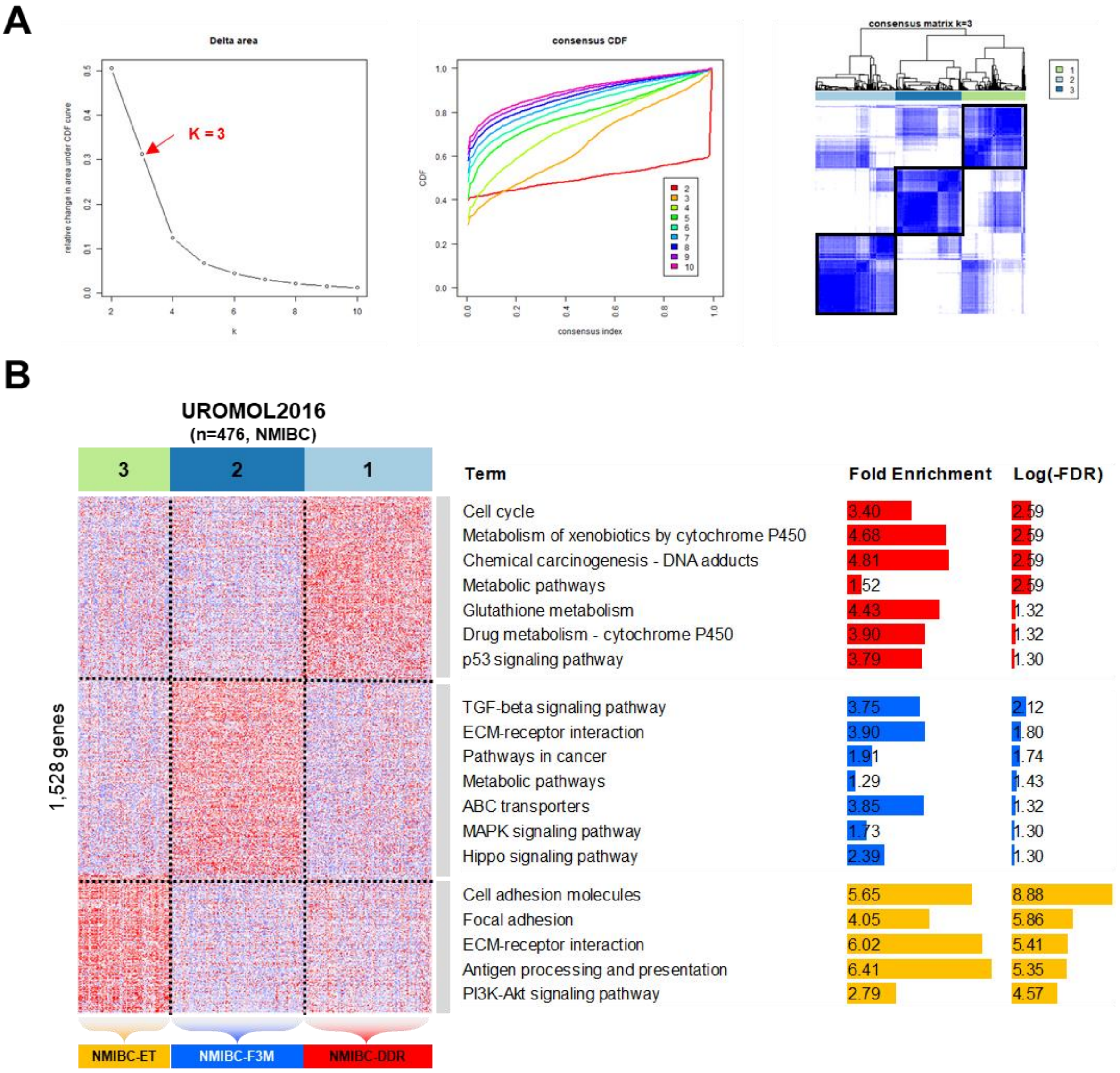

Supplementary Figure S1. Identification of the number of clusters through consensus clustering analysis and characterization of subtype through enriched biological pathway. (A) Cluster stability estimated according to relative changes in the area under the consensus distribution function curve across k = 2-10, revealed that 3 clusters were distinguishable in the NMIBC. Consensus matrix heatmap for k = 3. Data are presented in matrix format, in which white and blue denote no correlation (0) or a perfect correlation (1). (B) Gene expression patterns and enriched biological characteristics at five clusters. The color in the heatmap reflect relatively high (red) and low (blue) expression. Functional analyzes by DAVID representing defined clusters were indicated by colors and selected by statistical significance (FDR < 0.25).

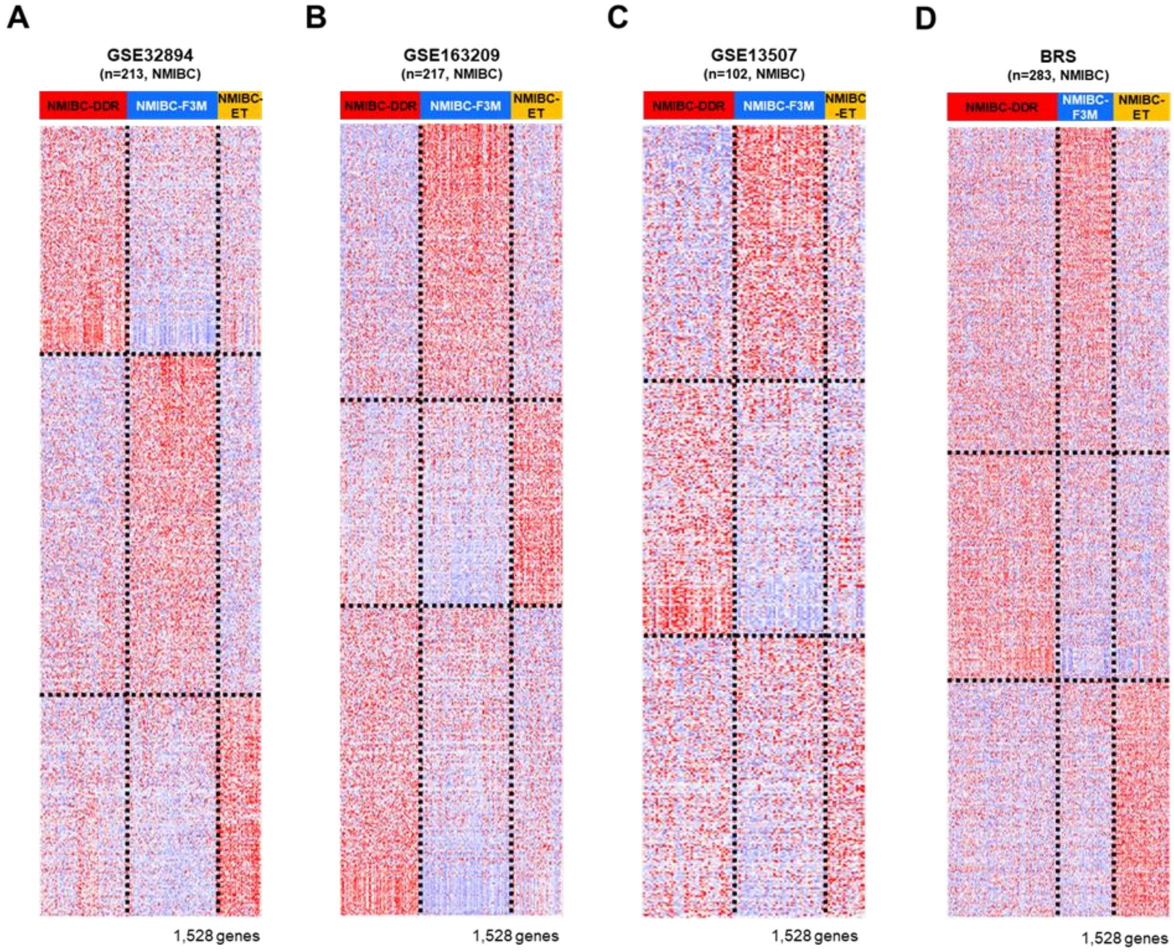

Supplementary Figure S2. Estimation of prognostic relevance in validation cohorts (NMIBC) based on the prediction models. (A, B, C, and D) Gene expression patterns of validation cohorts. The color in the heatmap reflect relatively high (red) and low (blue) expression.

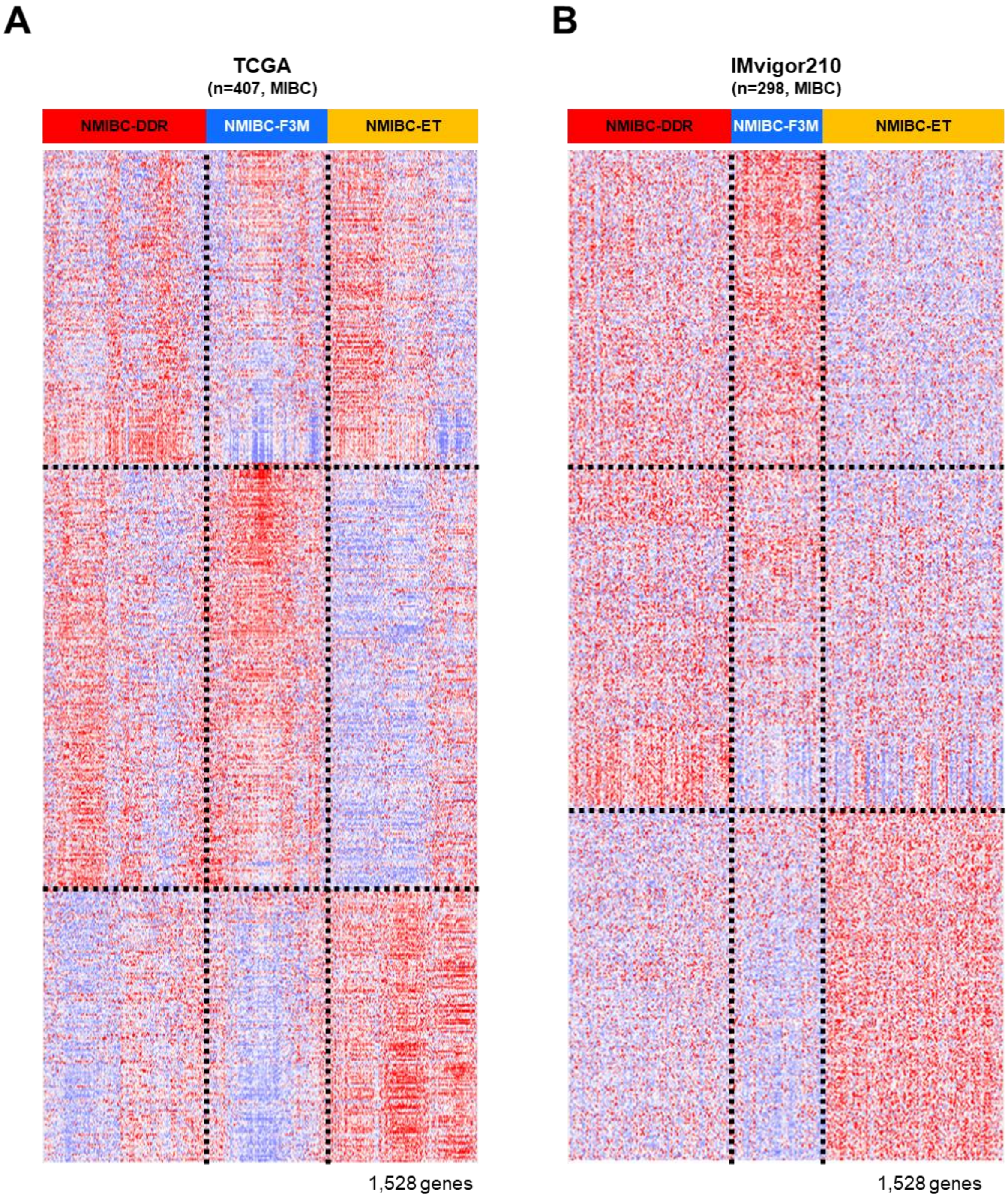

Supplementary Figure S3. Estimation of prognostic relevance in MIBC patients based on the prediction models. (A) Gene expression patterns of TCGA. The color in the heatmap reflect relatively high (red) and low (blue) expression. (B) Gene expression patterns of IMvigor210 trial with immunotherapy treatment. The color in the heatmap reflect relatively high (red) and low (blue) expression.

Supplementary Table S1. Results of grid search for hyperparameter modification

Rank	m_{try}	Node size	Sample size	OOB RMSE
1	30	2	0.8	0.268982
2	30	4	0.8	0.268982
3	10	6	0.8	0.273743
4	190	2	0.8	0.278423
5	30	6	0.8	0.278423
6	190	4	0.8	0.283025
7	140	6	0.8	0.283025
8	290	6	0.8	0.283025
9	30	8	0.8	0.283025
10	140	8	0.8	0.283025
11	10	12	0.8	0.283025
12	30	12	0.8	0.283025
13	60	12	0.8	0.283025
14	10	14	0.8	0.283025
15	140	14	0.8	0.283025
16	60	16	0.8	0.283025
17	110	4	0.8	0.287554
18	70	8	0.8	0.287554
19	10	10	0.8	0.287554
20	70	10	0.8	0.287554

Mtry, the number of variables to randomly sample as candidates at each split; OOB RMSE, Out of bag root-mean-square error

Supplementary Table S2. Result of retraining of prediction model by applying hyperparameters for improved performance

		NMIBC-DDR	NMIBC-F3M	NMIBC-ET
Prediction	**NMIBC-DDR**	145	0	0
	**NMIBC-F3M**	0	140	0
	**NMIBC-ET**	0	0	102

**Overall statistics (Training data)**	
Accuracy	1
Kappa	1
95% CI	(0.9905, 1)
P-value [ACC > NIR]	< 2.2E-16
Mcnemar's Test P-value	na

		NMIBC-DDR	NMIBC-F3M	NMIBC-ET
Prediction	**NMIBC-DDR**	29	2	0
	**NMIBC-F3M**	2	32	1
	**NMIBC-ET**	0	0	23

**Overall statistics (Test data)**	
Accuracy	0.9438
Kappa	0.9147
95% CI	(0.8737, 0.9815)
P-value [ACC > NIR]	< 2.2E-16
Mcnemar's Test P-value	na

Supplementary Table S3. Univariate and multivariate Cox proportional hazards regression analysis in the independent cohorts.

	**Variable**	**Univariate**				**Multivariate**			
		**hazardRatio**	**ciLower**	**ciUpper**	***p*-value**	**hazardRatio**	**ciLower**	**ciUpper**	***p*-value**
**Disease progression in the GSE163209 cohort**	Gender								
	Male (Ref.) vs. Female	0.54	0.18	1.66	0.289				
	Age								
	≤ 69 (Ref.) vs. > 69	2.81	1.01	7.78	0.04				
	Stage								
	Ta (Ref.) vs. T1	19.7	2.64	148.03	0.003	5.68	0.75	42.93	0.09
	Grade								
	Low (Ref.) vs. High	3.64E+08	0	inf	0.99				
	The multi-gene signature								
	Other classes (Ref.) vs. NMIBC-DDR	3.36	1.34	8.45	0.009	1.66	0.62	4.39	0.306

	**Variable**	**Univariate**				**Multivariate**			
		**hazardRatio**	**ciLower**	**ciUpper**	***p*-value**	**hazardRatio**	**ciLower**	**ciUpper**	***p*-value**
**Disease progression in the GSE32894 cohort**	Gender								
	Male (Ref.) vs. Female	0.56	0.16	1.95	0.364				
	Age								
	≤ 69 (Ref.) vs. > 69	2.42	0.86	6.84	0.095				
	Stage								
	Ta (Ref.) vs. T1	5.13	1.69	15.6	0.003	3.45	1.03	11.47	0.043
	Grade								
	Low (Ref.) vs. High	5.12	1.92	13.68	< 0.001				
	The multi-gene signature								
	Other classes (Ref.) vs. NMIBC-DDR	4.23	1.51	11.89	0.006	2.55	0.83	7.76	0.099

	**Variable**	**Univariate**				**Multivariate**			
		**hazardRatio**	**ciLower**	**ciUpper**	***p*-value**	**hazardRatio**	**ciLower**	**ciUpper**	***p*-value**
**Disease progression in the GSE13507 cohort**	Gender								
	Male (Ref.) vs. Female	0	0	inf	0.997				
	Age								
	≤ 69 (Ref.) vs. > 69	2.82	0.81	9.89	0.104				
	Stage								
	Ta (Ref.) vs. T1	1.11	0.29	4.25	0.877	0.62	0.13	2.81	0.541
	Grade								
	Low (Ref.) vs. High	3.00	0.77	11.68	0.112				
	The multi-gene signature								

	Other classes (Ref.) vs. NMIBC-DDR	9.64	1.99	46.77	0.004	8.64	1.56	47.79	0.013

	Variable	Univariate				Multivariate			
		**hazardRatio**	**ciLower**	**ciUpper**	***p*-value**	**hazardRatio**	**ciLower**	**ciUpper**	***p*-value**
**Overall survival in the TCGA cohort**	Gender								
	Male (Ref.) vs. Female	1.11	0.80	1.54	0.518				
	Age								
	≤ 69 (Ref.) vs. > 69	1.55	1.15	2.08	0.003	1.57	1.16	2.11	0.002
	Stage								
	Ta (Ref.) vs. T1	9205052	0	inf	0.992				
	Grade								
	Low (Ref.) vs. High	2.90	0.71	11.73	0.135				
	The multi-gene signature								
	Other classes (Ref.) vs. NMIBC-DDR	1.41	1.05	1.89	0.021	1.43	1.07	1.93	0.015
